# Increasing incidence and prevalence of Hodgkin’s lymphoma in Finland: a population-based registry study

**DOI:** 10.1093/eurpub/ckaf002

**Published:** 2025-01-20

**Authors:** Tessa Antikainen, Noora Hannuksela, Anna Anttalainen, Anu Partanen, Aino Rönkä, Hanne Kuitunen, Liisa Ukkola-Vuoti, Iiro Toppila, Tatu Miettinen, Outi Kuittinen

**Affiliations:** Faculty of Health Medicine, School of Medicine, Institute of Clinical Medicine Oncology, University of Eastern Finland, Kuopio, Finland; Faculty of Health Medicine, School of Medicine, Institute of Clinical Medicine Oncology, University of Eastern Finland, Kuopio, Finland; Medaffcon Oy, Espoo, Finland; Department of Medicine, Kuopio University Hospital, Kuopio, Finland; Faculty of Health Medicine, School of Medicine, Institute of Clinical Medicine Oncology, University of Eastern Finland, Kuopio, Finland; Kuopio University Hospital Cancer Center, Kuopio, Finland; Cancer Center, Oulu University Hospital, Oulu, Finland; Medaffcon Oy, Espoo, Finland; Medaffcon Oy, Espoo, Finland; Takeda Oy, Helsinki, Finland; Faculty of Health Medicine, School of Medicine, Institute of Clinical Medicine Oncology, University of Eastern Finland, Kuopio, Finland; Kuopio University Hospital Cancer Center, Kuopio, Finland

## Abstract

Hodgkin’s lymphoma (HL) is a lymphoid malignancy with an emphasized incidence in developed countries. This study aimed to assess the changes in the epidemiology of HL in Finland at the population level by utilizing data from six nationwide healthcare registries. A total of 2912 patients with HL, diagnosed and treated between 2000 and 2019 were matched by controls and divided into younger (<50 years) and older cohorts (≥50 years) for analysis. A slightly increasing trend in incidence per age group was observed. For the younger patients, the mean annual incidence was 3.19 for males and 2.89 for females. For the older patients, it was 3.07 and 1.59, respectively. Finland has higher incidence rates than other Scandinavian countries possibly due to unique human leucocyte antigen allele distribution. There was a notable increase in prevalence. For females, this was particularly emphasized between the ages of 30–50 years, while among males, it was more evenly distributed across all ages. As a result of improved disease management, the proportion of HL survivors is increasing.

## Introduction

Lymphomas are a heterogeneous group of cancers, classified into more than hundred subtypes according to the WHO-HAEMV5 classification [1]. Hodgkin’s lymphoma (HL) accounts for ∼13.5% of all lymphomas. In 2020, 83 000 new HL cases were diagnosed worldwide [2]. Although HL is rare, it is the most common cancer among adolescents. A bimodal incidence pattern is typical for HL, with the first peak observed in adolescents and young adults, and the second peak in older adults [3].

Globally, the age-standardized incidence of all lymphomas has increased [4], while it has remained comparatively stable for HL [5]. The incidence is higher in developed countries, such as Europe, Australia, and North America compared to developing countries. The mortality rate of HL has decreased in high-income countries due to its advanced treatment [5]. Currently, ∼90% of patients with HL are long-term survivors [6].

Prior epidemiological studies have focused on the incidence of HL; however, limited data are available on prevalence of HL and trends in occurrence over time. Reporting prevalence and incidence is significant since survivors of HL may be at risk for late morbidity [7]. This study aimed to address this issue by evaluating the population-level trends of HL prevalence and incidence in Finland. Additionally, we were interested in whether there was seasonal variation in the incidence.

## Methods

### Patients and methods

The patient cohort included 2912 incident patients with a record of HL (the International Classification of Diseases, Tenth Revision; ICD-10-code C81*) diagnosis in the Finnish Cancer Registry (FCR). Patients were diagnosed between 1 January 2000 and 31 December 2019 in Finland and divided into two patient cohorts: the younger (age <50 years at diagnosis) and the older (age ≥50 years at diagnosis) cohorts. A 1:1-ratio age, sex, and region of Finland matched control population was created from the Digital and Population Data Services Agency (DPA). A control was considered eligible for the control cohort if he/she was alive without a lymphoma diagnosis between 1 January 2000 and 31 December 2019 at the index of the corresponding case. In the corresponding cases, the controls were followed up based on the lymphoma index (lymphoma diagnosis). For <5 patients, a match with the same home municipality could not be found, therefore patients received controls without a regional match.

The younger cohort included 1855 patients, of whom 53.6% (994 patients) were male. The older patient cohort included 1057 patients, of whom 65% (661 patients) were male. For prevalence calculations, we included all patients who had been diagnosed with HL and were alive in the index year. Incidence data included all patients with a first diagnosis of HL during the study period.

### Materials

This retrospective registry study utilized data collected from six Finnish healthcare registries: the FCR (HL diagnosis), National Institute of Health and Welfare (HL diagnosis, treatments), the Social Insurance Institution of Finland and the Finnish Centre for Pensions (daily sickness allowance, pension, etc. resources), Statistics Finland (socioeconomical status), and Digital and Population Data Services Agency (gathering of controls). These were nationwide registries, as such, the data covered the entire population of Finland (∼5.6 million in 2019). The Finnish Social and Health Data Permit Authority (Findata, permit no. THL/1541/14.02.00/2021) and Statistics Finland (data permit no. TK/3616/07.03.00/2021) granted permission for data collection. Prevalence data were extracted from the FCR by using ICD-10 code C81.

### Statistical analyses

Using only existing data retrieved from the registries, analyses were performed using R software version 4.0.3. Missing values were not imputed. Statistical significance was set at *P* < .05. The tests were two-sided unless otherwise mentioned. All results and *P*-values were descriptive, and multiple testing corrections were not applied. All analyses were performed separately for the younger (<50 years) and the older (≥50 years) cohorts.

### Incidence and prevalence

Annual incidence, age-standardized annual incidence, and incidence by age group were calculated. The age-standardized annual incidence was scaled by the global distribution by the WHO [8]. The incidence and prevalence are presented as the number of annual cases per 100 000 persons.

Monthly incidences, which took into account month-length, were scaled to correspond to the annual incidence per 100 000 persons.

### Demographic and clinical characteristics

Demographic characteristics and the Charlson Comorbidity Index (CCI) were evaluated at the index. Sociological variables (e.g. number of biological children, educational level, and socioeconomic status) were evaluated using the most recent data available before diagnosis. CCI was calculated based on comorbidities and co-diagnoses, which, compared to baseline, had been recorded during 5 years prior to HL diagnosis. The CCI was calculated from the original scoring system [9] using the modified and updated scoring system [10]. Before calculating the CCI, lymphoma diagnoses (ICD-10 codes C81–C85) were excluded to avoid bias in the indices.

Median and first and third quartile (interquartile range) were reported for continuous variables. Categorical variables were reported as numbers and percentages. Traditional tests were used to assess the differences between patients and cohorts. The chi-squared test or Fisher’s exact test was used for categorical variables and the Kruskal–Wallis test was used for continuous variables. Paired tests were not performed because of the small sample sizes of some subgroups. No multiple test correction was applied.

## Results

### Demographics characteristics

The demographic characteristics of patients and controls are presented in Table 1. In the younger patient cohort, statistically significant differences between the patients and controls were detected in the CCI; CCI zero was detected for 96.6% of the controls, compared to 91.5% of the patients. CCI was one or over for 3.4% of the controls and 8.5% of the cases. Among the older patient cohort statistically significant differences were detected in the CCI and marital status. A total of 22.7% of patients had a CCI of one or two, and 10.5% had a CCI three or over. In comparison, 15.0% of the controls had a CCI of one or two and 3.7% had a CCI three or over. Among the older case cohort, more people were divorced (12.8%), widowed (9.9%), and unmarried (17.4%) compared to the controls (12.0%, 8.2%, and 14.0%, respectively).

**Table 1.** Demographic and clinical characteristics of the younger (<50 years) and older cohort (≥50 years)^a^

		Younger cohort				Older cohort			
		**HL patients** ***n* = 1855**	**Controls** ***n* = 1855**	*P*-value	Missing (%)	**HL patients** ***n* = 1057**	**Controls** ***n* = 1057**	*P*-value	Missing (%)
**Age at diagnosis, years, mean (SD)**		28.41 (9.79)	28.41 (9.79)	1.000		67.41 (10.48)	67.41 (10.48)	1.000	
**Male, *n* (%)**		994 (53.6)	994 (53.6)	1.000		661 (62.5)	661 (62.5)	1.000	
**Charlson comorbidity index, *n* (%)**	0	1698 (91.5)	1792 (96.6)	<.001		706 (66.8)	859 (81.3)	<.001	
	1 to 2	127 (6.8)	63 (3.4)			240 (22.7)	159 (15.0)		
	3 or over	30 (1.6)	0 (0.0)			111 (10.5)	39 (3.7)		
**Biological children, *n* (%)**	0	1132 (67.3)	1110 (65.7)	.120	9.2	104 (11.3)	93 (10.0)	.242	12.4
	1	174 (10.4)	192 (11.4)			167 (18.2)	171 (18.3)		
	2	225 (13.4)	261 (15.5)			334 (36.3)	362 (38.8)		
	3 or over	150 (8.9)	126 (7.5)			315 (34.2)	306 (32.8)		
**Marital status, *n* (%)**	Married or in a registered partnership	235 (13.3)	235 (13.3)	.119		608 (59.8)	667 (65.8)	.035	4.0
	Divorced	23 (1.3)	39 (2.2)			130 (12.8)	122 (12.0)		
	Widowed	–	–			101 (9.9)	83 (8.2)		
	Unmarried	1513 (85.4)	1493 (84.5)			177 (17.4)	142 (14.0)		
**Family type, *n* (%)**	Mother with children	181 (10.8)	191 (11.3)	.663	9.6	43 (5.5)	34 (4.2)	.272	24.6
	Married couple without children	60 (3.6)	53 (3.1)			336 (42.8)	349 (43.1)		
	Married couple with children	976 (58.4)	948 (56.3)			267 (34.0)	312 (38.6)		
	Cohabiting couple without children	220 (13.2)	253 (15.0)			68 (8.7)	58 (7.2)		
	Cohabiting couple with only non-common children	43 (2.6)	51 (3.0)			17 (2.2)	12 (1.5)		
	Cohabiting couple with children	155 (9.3)	154 (9.1)			32 (4.1)	29 (3.6)		
	Father with children	36 (2.2)	34 (2.0)			22 (2.8)	15 (1.9)		
**Education, *n* (%)**	Upper secondary	1010 (83.6)	983 (84.4)	.729	36.0	284 (55.1)	329 (58.1)	.781	48.9
	Post-secondary non-tertiary	<5	<5			<5	<5		
	Short-cycle tertiary	89 (7.4)	82 (7.0)			118 (22.9)	121 (21.4)		
	Bachelor’s degree or equivalent	57 (4.7)	46 (3.9)			53 (10.3)	62 (11.0)		
	Master’s degree or equivalent	47 (3.9)	44 (3.8)			53 (10.3)	46 (8.1)		
	Doctorate or equivalent	<5	6 (0.5)			6 (1.2)	6 (1.1)		
**Sosioeconomic status, *n* (%)**	Lower-level employee with administrative and clerical occupations	402 (23.5)	367 (21.8)	.293	8.4	141 (14.2)	154 (15.6)	.832	6.1
	Pensioner	27 (1.6)	22 (1.3)			410 (41.2)	389 (39.3)		
	Students	10 (0.6)	17 (1.0)			6 (0.6)	5 (0.5)		
	Other	281 (16.4)	275 (16.3)			188 (18.9)	193 (19.5)		
	Manual worker	509 (29.7)	487 (28.9)			68 (6.8)	75 (7.6)		
	Unemployed	124 (7.2)	157 (9.3)			84 (8.4)	89 (9.0)		
	Upper-level employees with administrative, managerial, professional, and related occupations	230 (13.4)	224 (13.3)			99 (9.9)	85 (8.6)		
	Self-employed	129 (7.5)	137 (8.1)			141 (14.2)	154 (15.6)		

a Small patient groups (<5 patients) were not reported in detail.

### Absolute numbers

The total number of patients diagnosed with HL was 2912 between 2000 and 2019. A total of 1655 patients (56.8%) were male. Of the patients, 1855 (63.7%) were under the age of 50 years and 1057 (36.3%) were 50 years or older. Of those under 50 years, 994 (53.6%) were male, and of those over 50 years, 661 (62.5%) were male.

### Annual incidence

The mean annual incidence between 2000 and 2019 was 2.72 per 100 000. The annual incidence increased between 2000 and 2019 from 2.35 per 100 000 to 3.22 per 100 000 (*P* = .001). The mean age-standardized annual incidence between 2000 and 2019 was 2.68 per 100 000. The age-standardized incidence increased during the research period from 2.35 to 3.14 (*P* = .003). The mean age-standardized incidence for males and for females was 2.95 and 2.43 per 100 000 per year.

### Annual incidence according to sex and age group

**Figure 1.**
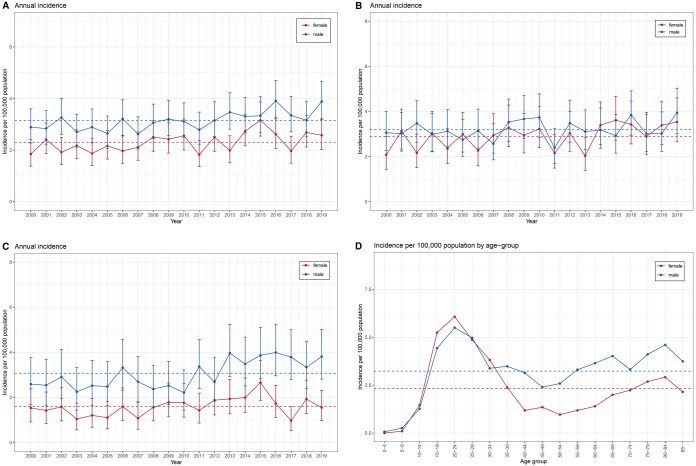
(A) The annual incidence of Hodgkin’s lymphoma by sex. (B) The annual incidence of Hodgkin’s lymphoma for patients <50 years by sex. (C) The annual incidence of Hodgkin’s lymphoma for patients ≥50 years by sex. (D) The annual incidence of Hodgkin’s lymphoma by age group and sex.

The annual incidence between 2000 and 2019 according to sex is presented in Fig. 1A. The mean incidence for males and females was 3.14 and 2.3 per 100 000 per year, respectively. Between 2000 and 2019, annual incidence increased for males from 2.89 to 3.89 and for females from 1.85 to 2.57 per 100 000, respectively. The annual incidence among males was slightly higher than that among females throughout the study period.

The annual incidence in the younger cohort according to sex is presented in Fig. 1B. The mean incidence in males was 3.19 per 100 000 per year, which was higher than that in women (2.88 per 100 000). Between 2000 and 2019, incidence has increased from 3.06 to 3.94 and 2.07 to 3.54 per 100 000 among males and females, respectively.

The annual incidence in the older cohort according to sex is presented in Fig. 1C. The mean incidence for males and females was 3.07 and 1.59 per 100 000 per year, respectively. Between 2000 and 2019, the incidence has increased from 2.59 to 3.81 and 1.53 to 1.55 per 100 000 for males and females. The difference in incidence rates between sexes was greater for the older cohort than the younger cohort.

Incidence had bimodal age distribution by sex (Fig. 1D).

### Monthly incidence

No statistically significant seasonal variation was seen in the incidence (Supplementary Material S1). Among younger patients, the incidence tended to be lowest in late summer to autumn (July to October) (*P* = .098), and among older patients in late winter to early autumn (February to September) (*P* = .208).

### Prevalence

**Figure 2.**
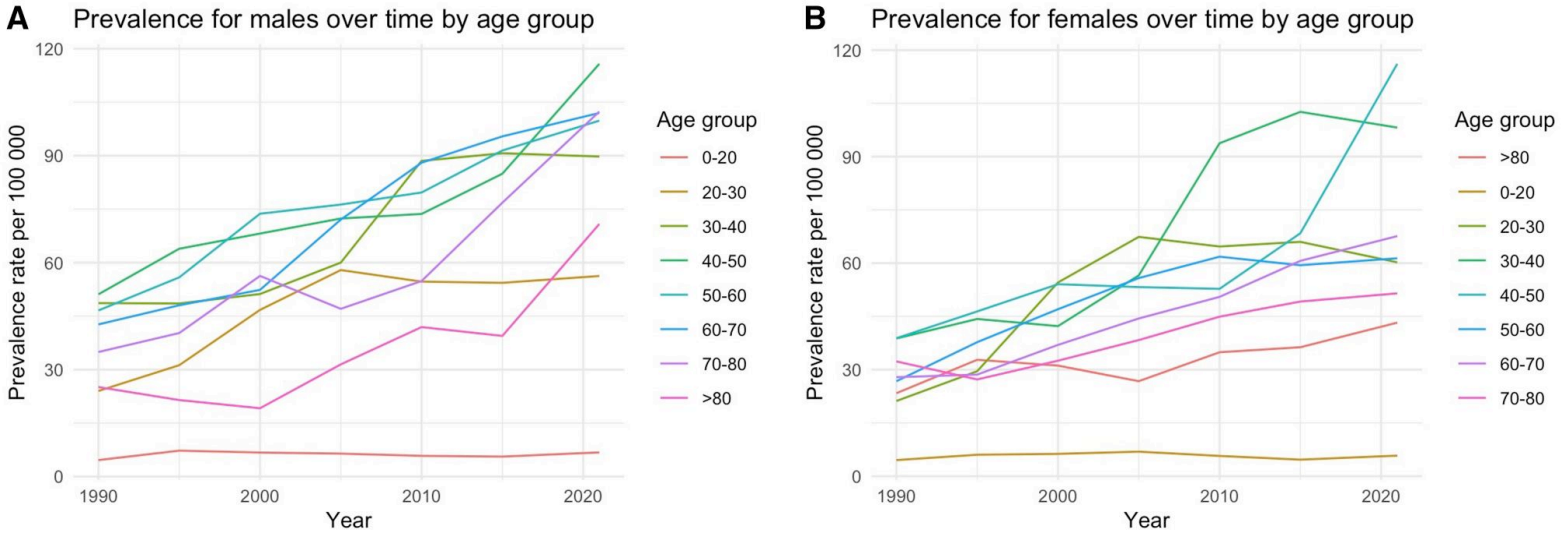
(A) The age-standardized prevalence of Hodgkin’s lymphoma for males by age group. (B) The age-standardized prevalence of Hodgkin’s lymphoma for females by age group.

The trends in HL prevalence between 1990 and 2021 are presented in Fig. 2. The prevalence is higher in males compared to females. In both males and females, the prevalence was higher in the older cohort compared to the younger cohort. The number of prevalent cases and age-standardized rates (ASR) by age group are shown in Table 2.

**Table 2.** Age-standardized prevalence rates and absolute cases numbers of Hodgkin’s lymphoma

	31 December 1990				31 December 2000				31 December 2010				31 December 2021			
	Male		Female		Male		Female		Male		Female		Male		Female	
Age group	*n*	ASR rate	*n*	ASR rate	*n*	Std rate	*n*	ASR rate	*n*	ASR rate	*n*	ASR rate	*n*	ASR rate	*n*	ASR rate
**0–20**	29	4.63	27	4.53	45	6.75	40	6.28	38	5.82	36	5.70	42	6.79	34	5.78
**20–30**	89	24.01	76	21.17	151	46.75	169	54.55	190	54.68	213	64.68	194	56.26	195	60.23
**30–40**	197	48.65	150	38.78	191	51.20	152	42.26	297	88.54	299	93.77	338	89.76	344	98.18
**40–50**	203	51.12	147	38.85	269	68.16	208	54.07	270	73.66	189	52.76	400	115.75	385	116.13
**50–60**	121	46.60	72	26.74	273	73.72	175	47.02	300	79.68	236	61.82	353	99.82	215	61.37
**60–70**	90	42.68	75	27.88	120	52.36	95	36.97	294	88.03	178	50.50	351	101.98	247	67.62
**70–80**	38	34.94	64	32.32	83	56.28	74	32.52	97	54.85	102	44.92	280	102.36	165	51.49
**>80**	10	25.11	25	23.36	10	19.17	40	31.14	35	41.93	61	34.90	87	70.84	89	43.24

*n*, number of cases; ASR rate, age-standardized prevalence rate per 100 000.

## Discussion

This study evaluated the trends in the incidence and prevalence of HL in the Finnish population between 2000 and 2019. Our study found two age peaks in the incidence of HL. The incidence rate increased slightly during the study period. We found a remarkable increase in the prevalence of up to two to three times in the oldest age group during study time.

HL is a rare type of cancer, accounting for 0.4% of all cancers and of ∼10% of all lymphomas. Its incidence is higher in males than in females [11]. The HL incidence pattern is bimodal, as incidence is highest among young adults and older adults (over 55 years). Owing to advanced treatment strategies in the last couple of decades, the prognosis of HL has improved. The outcome depends on age and disease stage. As many as 90% of young patients with early-stage disease patients survive, the corresponding survival rate is approximately 75%–90% in advanced-stage disease [12]. Survival rate is worse for older adults [13].

The aetiology of HL is incompletely understood, however, there are known risk factors. Genetic factors interfere with the aetiology of HL [14]. Some alleles of human leucocyte antigens (HLAs) are associated to have an impact of developing classical HL [15]. HL is associated with viral infections such as Epstein-Barr virus (EBV) [16] and human immunodeficiency virus (HIV) [17] as well as other immunosuppressive conditions [18]. In Europe, 35.5% and in North America, 31.8% of HL cases are EBV+ [19]. The impact of HIV infection on the increase in incidence is presumably low. Certain autoimmune diseases, such as rheumatoid arthritis, systemic lupus erythematosus and sarcoidosis, or a family history of sarcoidosis or ulcerative colitis are associated with HL [20]. The incidence rates are higher in countries with a high sociodemographic index (SDI) than in those with a lower SDI [5]. In general, HL incidence has been associated with high socioeconomic status, for which is typical small family sizes. This may have been caused by a delay in exposure to infectious agents [21]. In Finland, family sizes have decreased over time and the environment is hygienic which decreases exposure to infectious agents. In addition, an increase in risk factors, such as obesity, hypertension and smoking may have had an impact on the increase in incidence [22]. In our study, the incidence increased especially among those over 50 years, which may have been affected by an increase in the prevalence of these risk factors.

Based on our results, comorbidities were more common in patients with HL than in controls in both age groups. However, diagnostic bias cannot be excluded. Comorbidities are more likely to be diagnosed among patients with HL owing to closer medical examinations.

In 2020, the global HL age-adjusted incidence rate was 0.98 per 100 000 [22]. The incidence was highest in Europe [Southern (ASR = 2.8), Northern (ASR = 2.6), and Western (ASR = 2.5)], Australia, and New Zealand (ASR = 2.6). Trends of increasing incidence [22] were in line with our results.

In a global comparison [22], the HL incidence rates observed in the Scandinavian countries are high, according to data available on NORDCAN [23]. Furthermore, the incidence rates are higher in Finland compared to Sweden. In Sweden, between 2017 and 2021, the age-standardized incidence for males and females was 2.4 and 1.9 per 100 000, respectively. In Finland, the age-standardized incidence between 2000 and 2019 was for males 2.95 and for females 2.43 per 100 000. Moreover, in contrast to Finland, incidence has been decreasing in Sweden in the last 10 years (estimated annual chance for the last 10 years for males was −1% and for females was −1.7%). In addition, the incidence of other lymphomas was higher in Finland than in Sweden [23]. Finland and Sweden are similar high-income countries, family sizes are small, populations are scattered, and exposure to antigens in childhood is low. Nevertheless, there is an unexplained difference in the incidence trends between Finland and Sweden. One explanation for these differences might be the genetic constitution, because the Finnish population is one among the most genetically isolated populations in the world. Finland has different frequencies of HLA antigens and haplotypes than other European countries. HLA haplotypes in Finnish population are more homogenous compared to Sweden. Finland is more genetically distant from Sweden than Sweden is from Germany, e.g. [24].

The incidence of HL is lower in the USA than in Finland. In the USA, the age-adjusted incidence was 2.5 per 100 000 per year between 2016 and 2020, and decreased by ∼1.6% per year between 2010 and 2019 [25, 26]. Whereas in Finland, the trend has been increasing. The gradually decreasing incidence of HL in the USA may be partly explained by the decreased number of HIV infections [27]. However, contradictory results have been presented [28]; after antiretroviral treatment became available, the incidence of HL may have even increased. HL is also associated with a high human development index [22] and both the USA and Finland are high-income countries. The USA has a higher gross domestic product (GDP) per capita than Finland. However, in the US income inequality is high, and a higher GDP is not reflected in the standard of living at the population level.

A previous registry study showed that the incidence varies seasonally, especially at higher latitudes, with the incidence being higher in winter and lower in summer [29]. Similar trends were also observed in the present study. The seasonal variation maybe related to vitamin D levels, as lower incidence rates are observed in seasons when vitamin D levels are higher, but variation was not statistically significant. This indicates that vitamin D somehow may protect against HL. However, vitamin D levels of the subjects were not available in this study. In addition to this seasonality, EBV infection can also affect seasonal variations in incidence [29]. However, it is unclear whether infection is a cause or sign of HL, as patients are more susceptible to infections [30]. It should also be taken into account that diagnostic delays may interfere with these numbers. The lower incidence rates in summer may be due to patients’ delays in medical care. Seasonal variations were not as noticeable in older adults as in younger people, consistent with a previous study [29]. HL in older adults is histologically and biologically different from that in younger individuals. Their HL appears to be more aggressive, and patients have more B symptoms [31]. Other illnesses are more common among older adults, and they may have more medical contacts, which may have an impact on less seasonal variation than in younger patients.

Few studies have described the trends in the prevalence of HL [32, 33]. We found a remarkable increase in the prevalence of HL among middle-aged people. These were mostly people diagnosed during the late 80s and the early 90s at the time when treatment lines changed from MOPP (mechlorethamine, oncovin, procarbazine, and prednisone) and MOPP-ABVD to ABVD (doxorubicin, bleomycin, vinblastine, dacarbazine) therapy. Along with the adoption of autologous haematopoietic stem cell transplantation as salvage therapy, these have resulted in improved survival rates. In Finland, the 5-year survival rate of male and female patients improved from 67% to 90% and from 73% to 92%, respectively [34].

Unfortunately, HL treatment has toxic late effects, such as secondary malignancies and cardiovascular diseases, which cause excess mortality among long-term survivors. MOPP with large-field radiotherapy, MOPP/ABV with or without radiotherapy, BEACOPP (bleomycin, etoposide, doxorubicin, cyclophosphamide, procarbazine, and prednisone) increased the risk of secondary leukaemia and myelodysplastic syndrome, which have a poor prognosis after HL. Radiotherapy increases the risk of breast cancer. The larger the chest radiation field size or dose, the greater the risk. Alkylating chemotherapy, on the other hand, have protective effect against breast cancer. However, the risk of lung and gastrointestinal cancers increases after HL treatment. Large-dose radiotherapy to the mediastinum increases the risk of cardiovascular diseases such as coronary artery disease, valvular disease, pericardial disease, arrhythmia, and cardiomyopathy [7]. As the prevalence increases with improved therapies, more recovered people are at risk of these late effects, which may increase costs in public health care.

A limitation of this study is the lack of detailed treatment information. In addition, as a real-world evidence-based study, registry data and errors in recording may exist. However, these registries are, in general, comprehensive, and reliable. The strengths of our study include the possibility to combine multiple registries, high coverage of data collection and a long study period. Despite socioeconomic status, all permanent residents in Finland are entitled to public healthcare, thus the data are nationwide, covering the entire population in Finland. Each patient with a HL was matched to controls by age, sex, and region.

## Conclusions

In summary, the incidence of HL is high in Finland compared to global rates. The incidence of HL has increased slightly, whereas prevalence has increased considerably. The considerably increased prevalence has resulted in an increasing number of long-term HL survivors in Finland who require follow-up for late effects. Finland has a different incidence trend than the USA and Sweden, although there is no clear reason for this. The reason for the increase in incidence and the difference between the USA and Sweden require further investigations. We observed seasonal variation in incidence in Finland. Vitamin D’s protective role might explain this variation, however more studies are necessary to elucidate this variation.

## Supplementary Material

ckaf002_Supplementary_Data

## Data Availability

Data used in this study cannot be made public, according to Finnish laws and legislations.

## Supplementary data

Conflict of interest: A.P. reports honoraria from Behring and Abbvie and has participated in Scientific Advisory Board meetings organized by Abbvie, Janssen-Cilag, Novartis, Pfizer, and Takeda. A.R. reports personal fees for speaking at symposia or financial support for attending conferences from Amgen, Bristol-Myers Squibb, Merck, Novartis, Pfizer, Daichii Sankyo, and Roche. None of the financial support was related to the present study. T.M. has been contractor by Takeda Oy during the study time. L.U.-V., I.T., and A.A. are employed by Medaffcon Oy. All other authors declare no competing interests.
