# Supplementary material for: Increasing incidence and prevalence of Hodgkin’s lymphoma in Finland: a population-based registry study
Source: Eur J Public Health. 2025 Jan 20;35(3):456–62. doi: 10.1093/eurpub/ckaf002 (PMC12187456; doi:10.1093/eurpub/ckaf002)

Supplementary material S1

(A) The seasonal variation of incidence for patients <50 years. (B) The seasonal variation of incidence for patients ≥50 years.


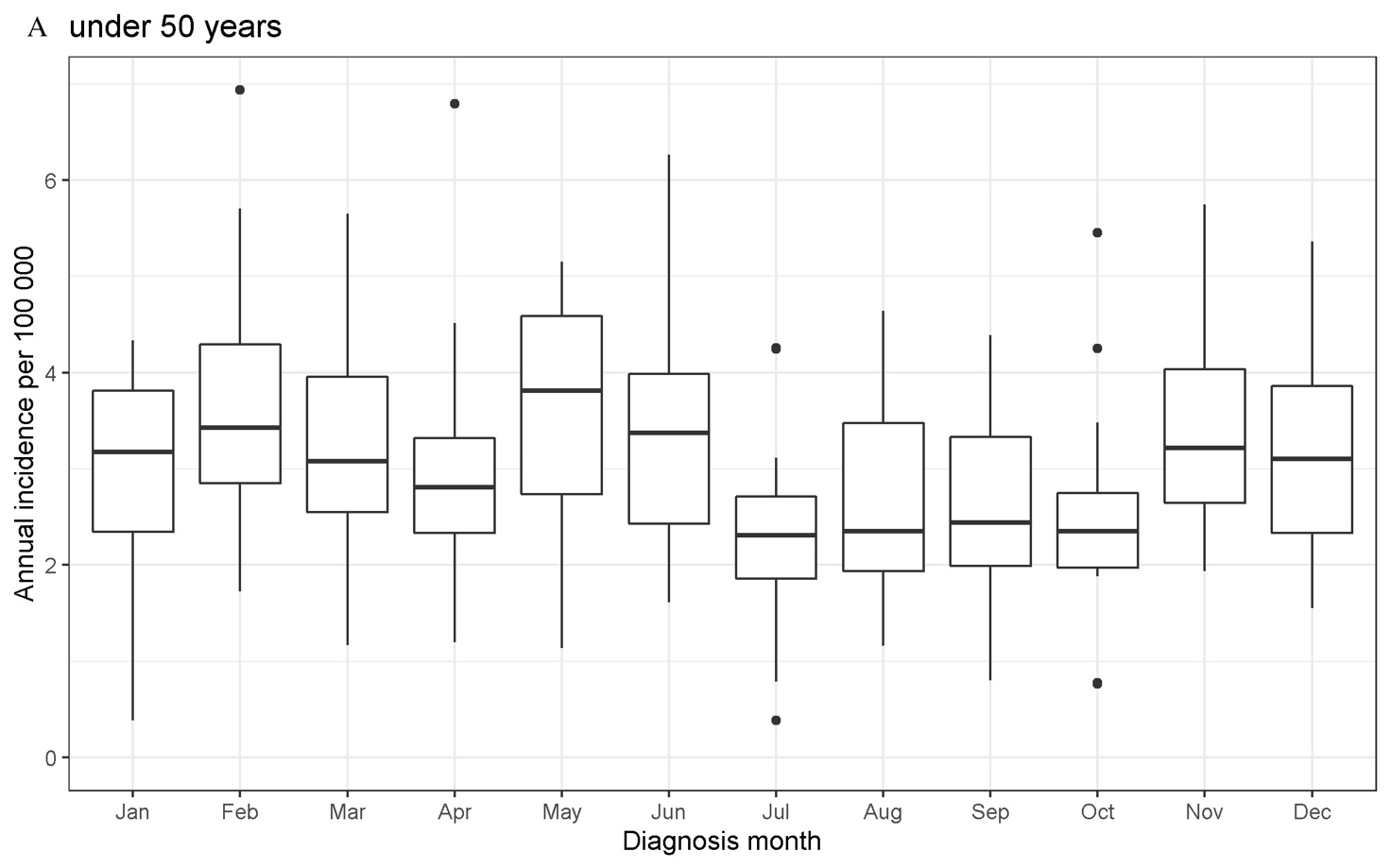


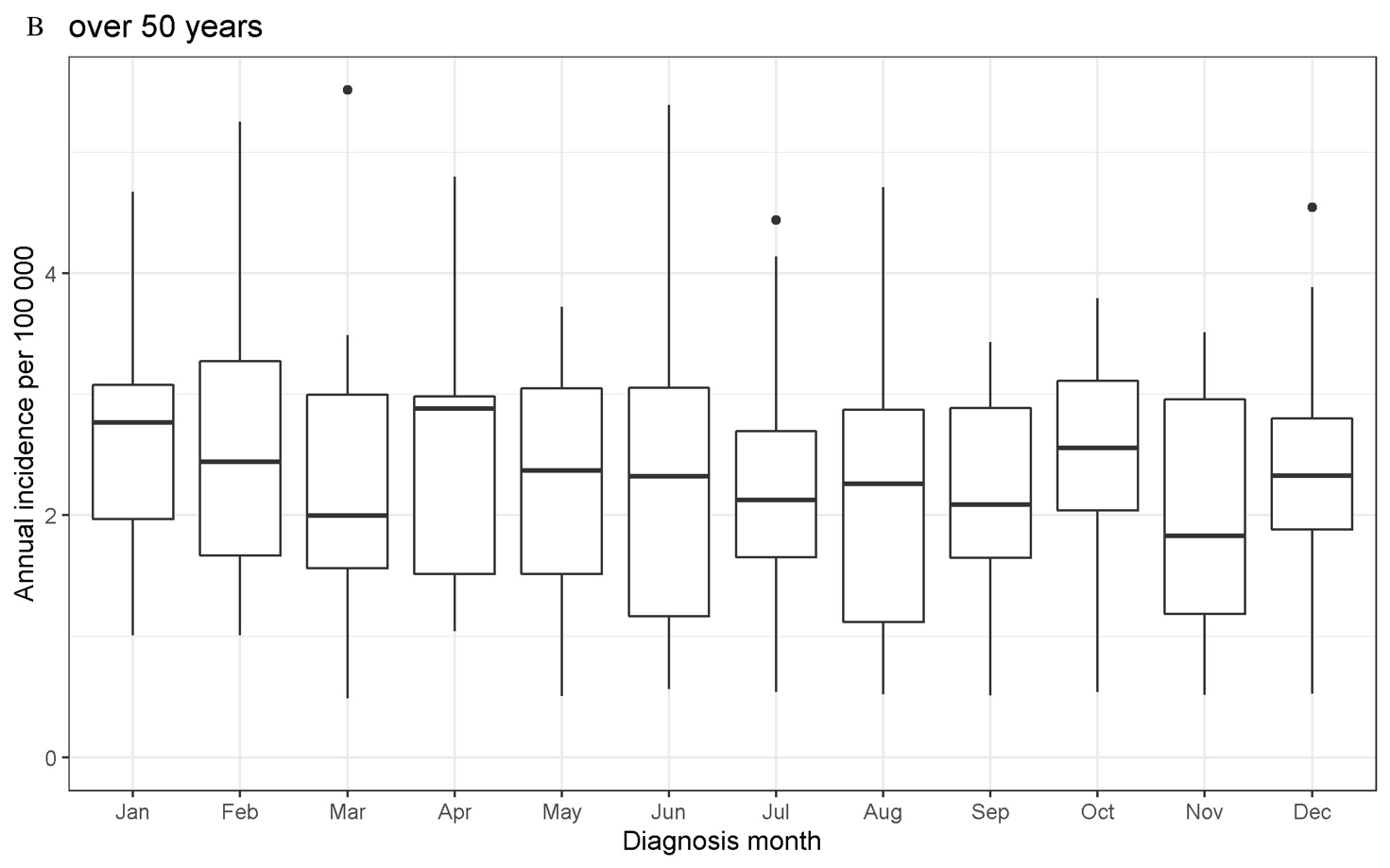

Supplement: ckaf002_Supplementary_Data [file ckaf002_supplementary_data.docx]
